# Supplementary material for: Lipidomic signatures align with inflammatory patterns and outcomes in critical illness
Source: Nat Commun. 2022 Nov 10;13:6789. doi: 10.1038/s41467-022-34420-4 (PMC9647252; doi:10.1038/s41467-022-34420-4)
Supplement: Supplementary file 1 — Supplementary Information [file 41467_2022_34420_MOESM1_ESM.pdf]

Supplementary Figures

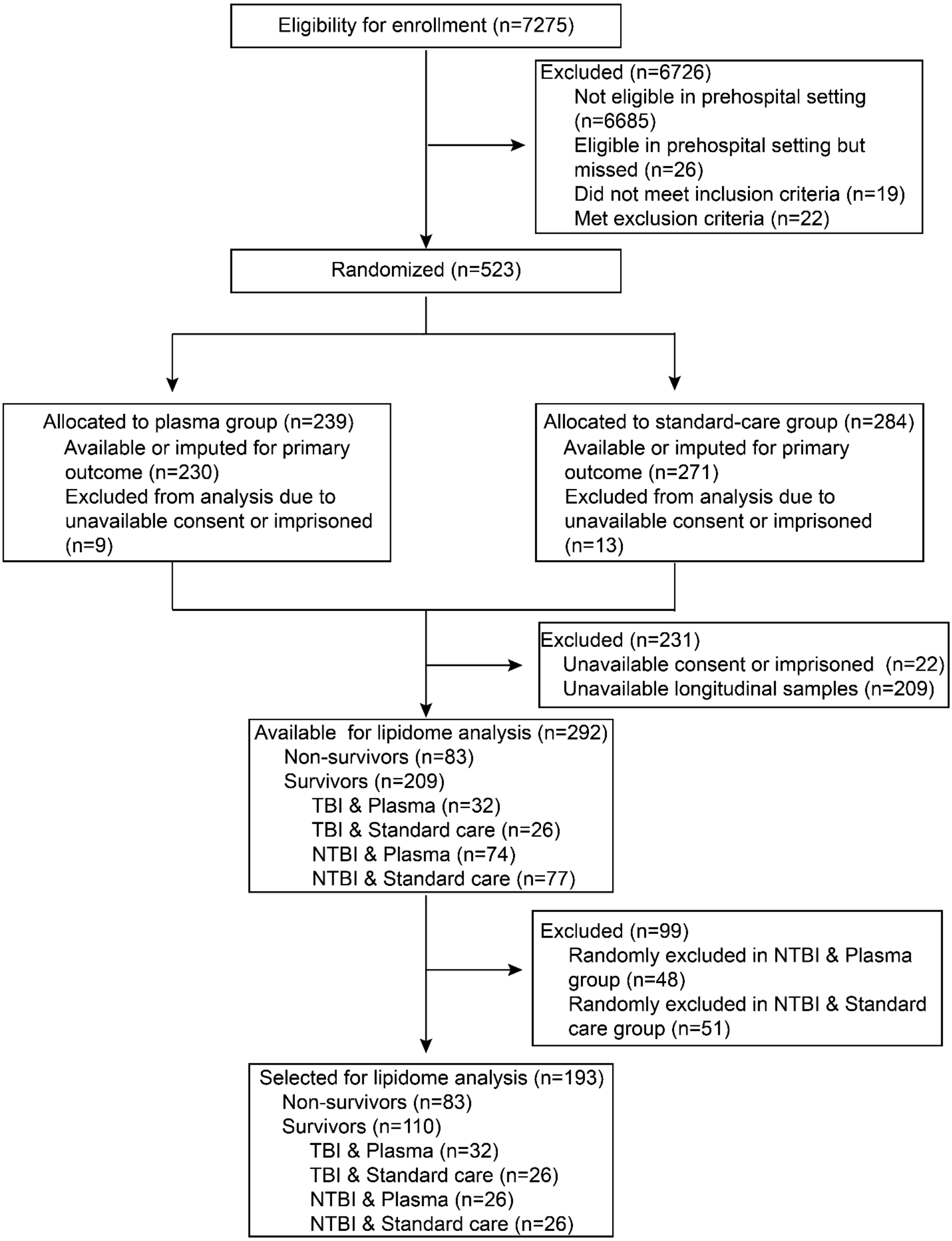

Supplementary Fig.1 Consort diagram. Screening, randomization and sampling for lipidomic analysis.

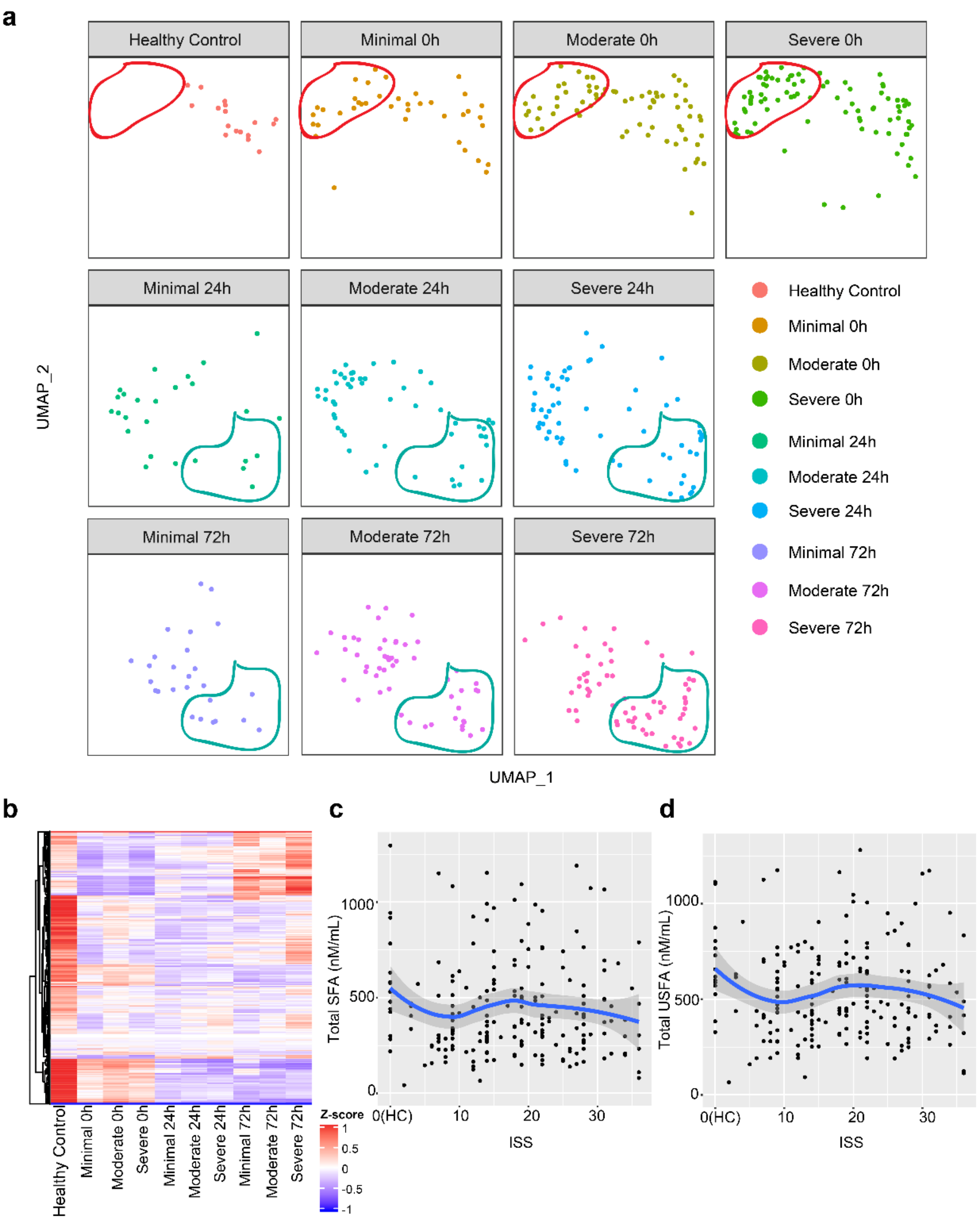

Supplementary Fig.2 Relationship of the circulating lipidome to injury severity.

(a) Uniform Manifold Approximation and Projection (UMAP) plot shows the distribution of healthy subjects (n=17) and patients with trauma (n=193), grouped by injury severity and sampling timepoints**.** (Minimal: ISS<10, Moderate: 10<=ISS<25, Severe: ISS>=25)

(b) Heatmap showing relative levels of 996 lipid species for healthy subjects and trauma patients, grouped by injury severity and sampling timepoints. Exp, z-score normalized concentration. Rows are clustered by hierarchical clustering.

(c-d) Relationship of ISS to absolute concentration of total saturated fatty acid (c) and unsaturated fatty acid (d) at 0h revealed by scatterplot. Error bands: 95% confidence interval for regression line.

ISS, injury severity score; SFA**:** saturated fatty acid; USFA: unsaturated fatty acid.

Supplementary Figures

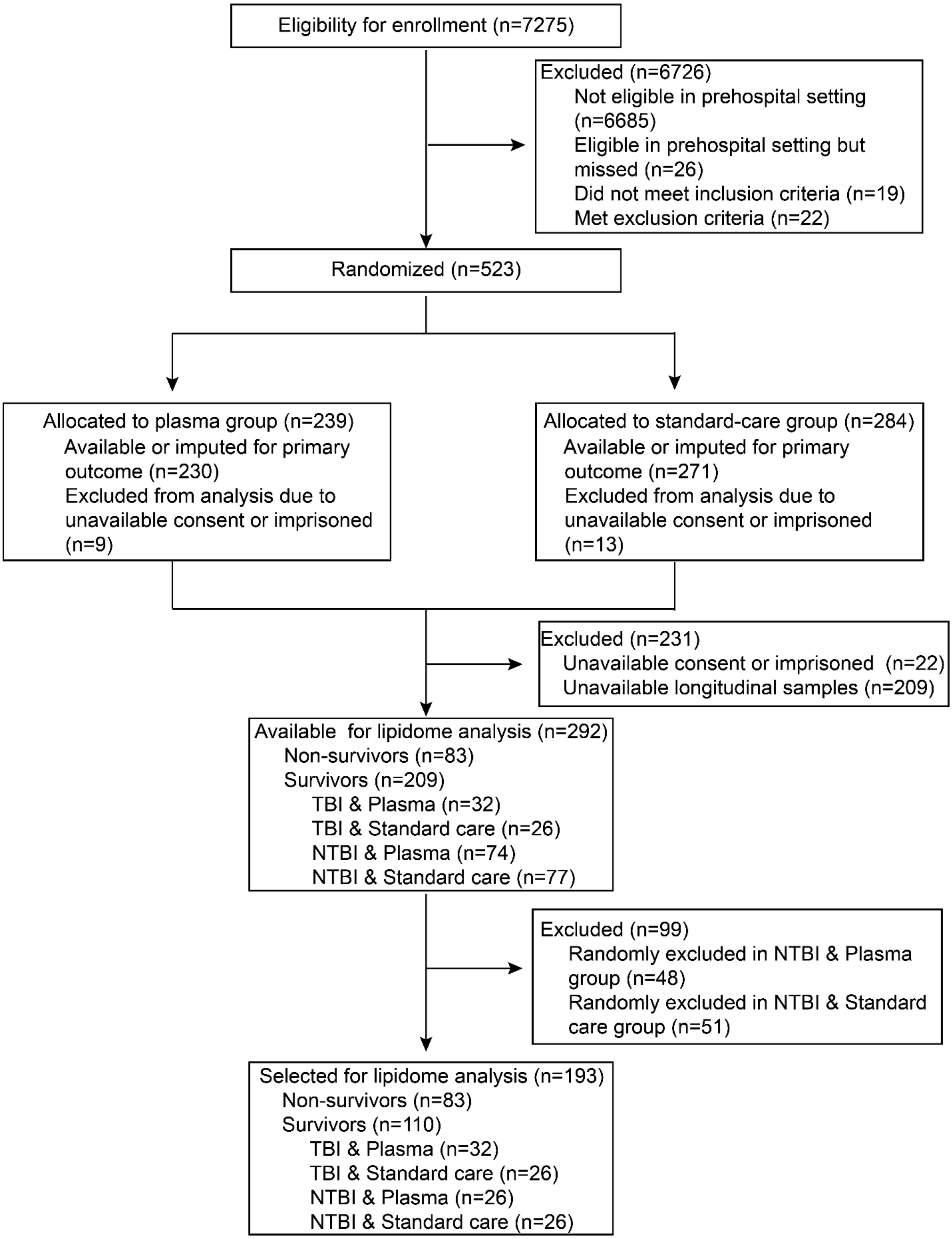

Supplementary Fig.1 Consort diagram. Screening, randomization and sampling for lipidomic analysis.

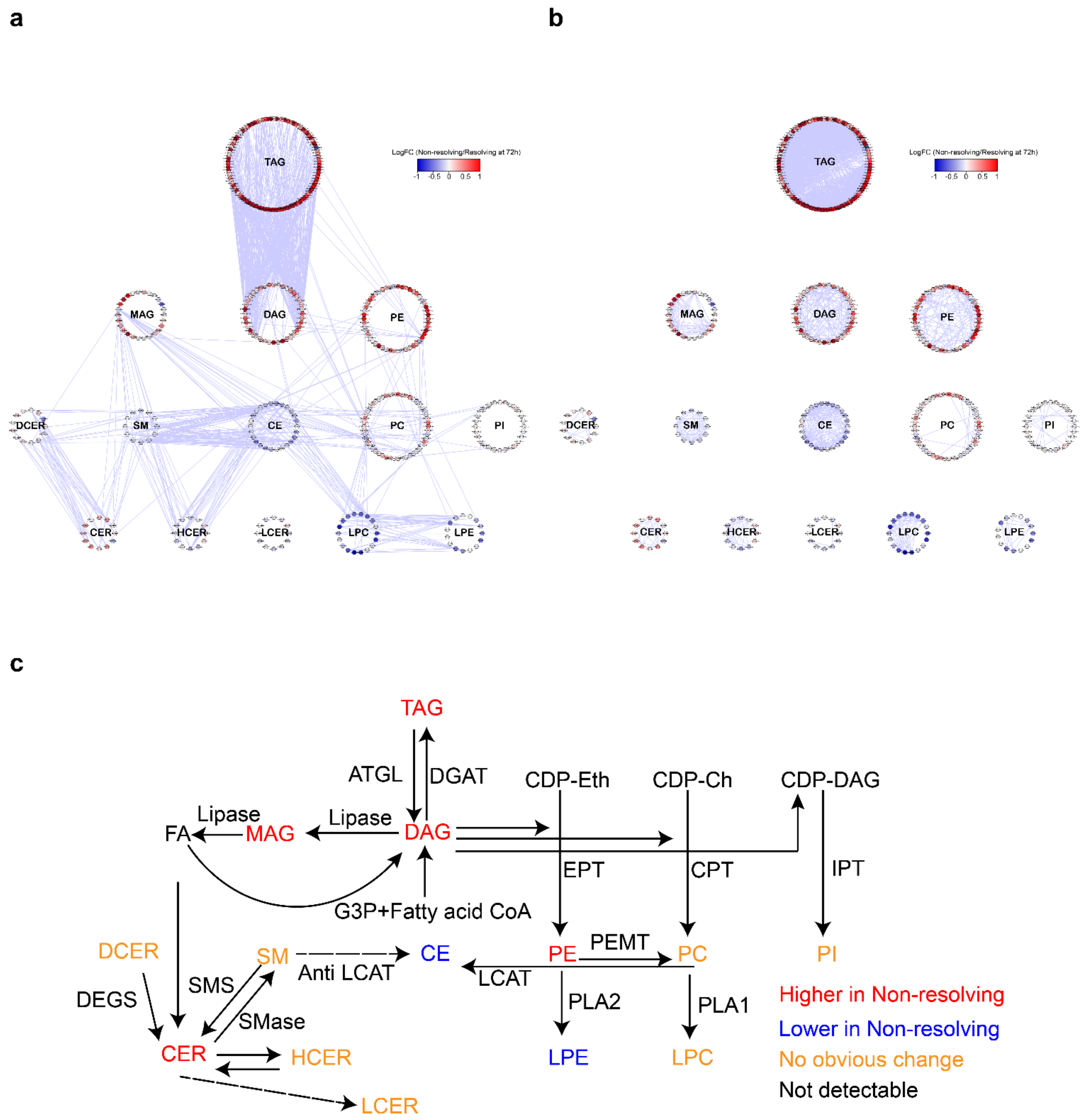

Supplementary Fig.3 Lipidome network in non-resolving trauma patients at 72h

(**a-b**) Inter-class(a) or Intra-class(b) Correlation network among 412 lipids from 14 classes represented in the lipidomic dataset. Each dot indicates a lipid and is depicted in a circle if it belongs to one class. Highly correlated (Pearson coefficient > 0.7) lipids are represented by edges. Relative levels are color coded for each lipid species between non-resolving (n=101) and resolving trauma patients (n=41) at 72h after admission.

(**c**) Synthesis pathways for the 14 lipid classes summarized from published literature. Colored by differential levels of each lipid class between non-resolving and resolving trauma patients at 72h admission.

Abbreviations: ATGL, Adipose Triglyceride Lipase; DAGT, diacylglycerol acyltransferase; G3P, glycerol-3-phosphate; CDP-Eth, Cytidine diphosphate-Ethanolamine; CDP-Ch, Cytidine diphosphate-Choline, CDP-DAG, Cytidine diphosphate-diacylglycerol, EPT, Ethanolamine phosphotransferase; CPT, Choline phosphotransferase; IPT, inositol phosphatidyltransferase. PLA, phospholipase A; PEMT, Phosphatidylethanolamine N-methyltransferase; LCAT, cholesterol acyltransferase; SMS, Sphingomyelin Synthase; SMase, Sphingomyelin phosphodiesterase; DEGS, dihydroceramide desaturase.

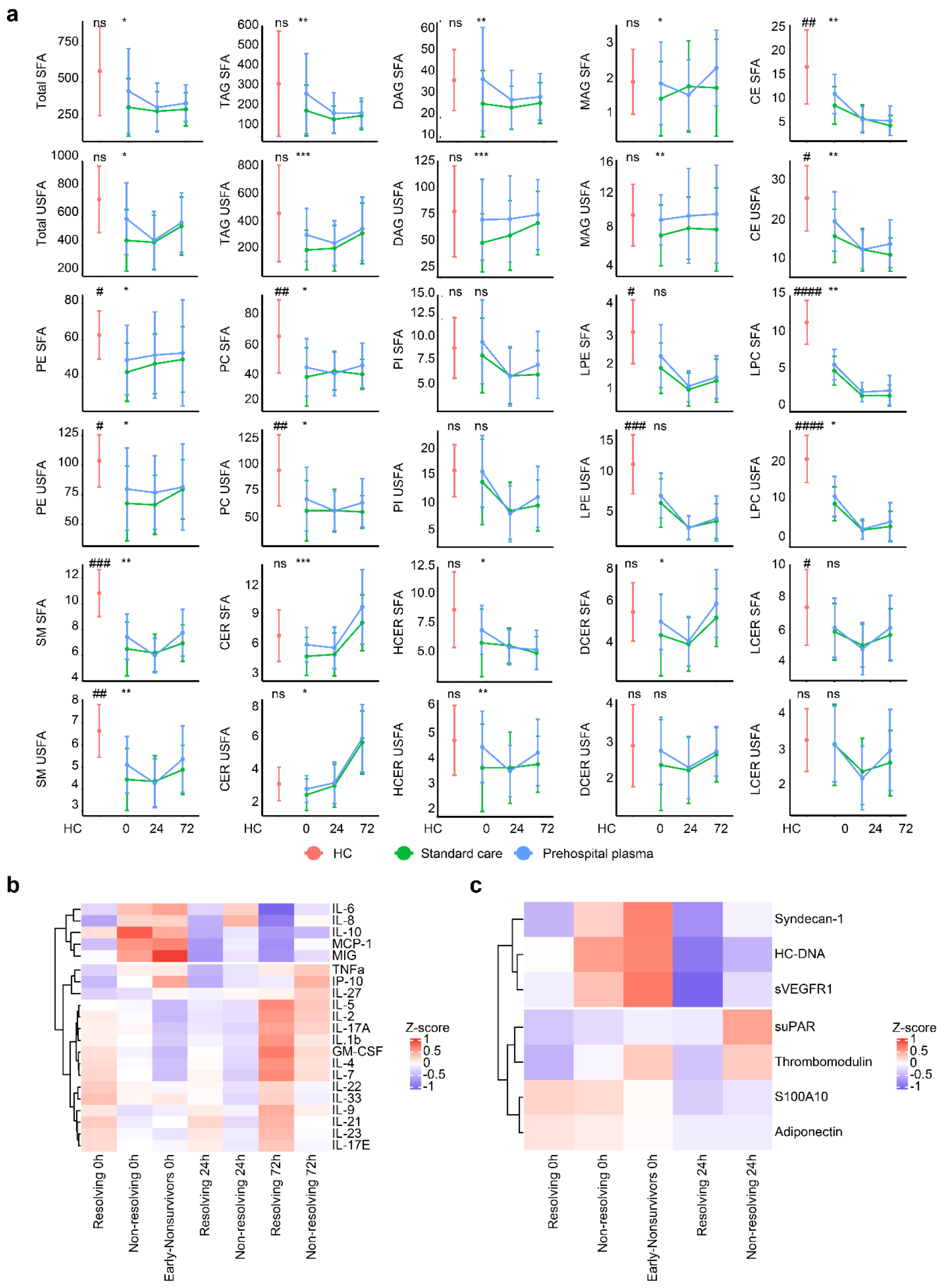

Supplementary Fig.4 Prehospital thawed allogeneic plasma (TP) can enhance levels of major lipid class

(a) Comparison of circulating total lipid concentration between standard care and prehospital TP. Lipids are grouped by classes and fatty acid (saturated or unsaturated) contained in the lipids. Patients (n=193) are grouped by treatment and sampling timepoints. Center dots and error bars represent median value and median absolute deviation, respectively. SFA**:** saturated fatty acid; USFA: unsaturated fatty acid. Asterisks indicate statistical significance between baseline and prehospital TP arm. Number sign indicates statistical significance between treatment arms in 0h. Kruskal-wallis test was used among baseline and treatment arms at 0h with post-hoc analysis of Dunn test. p value was adjusted by Benjamini-Hochberg method: *, < 0.05; **, < 0.01, ***, < 0.001; #, < 0.05; ##, < 0.01; ###, < 0.001, #### < 0.0001.

(b) Heatmap shows temporal pattern of circulating cytokines in trauma patients at 0h,24h and 72h after admission.

(c) Heatmap shows temporal pattern of circulating biomarkers in trauma patients at 0h and 24h after admission.

Source data are provided as a Source Data file.

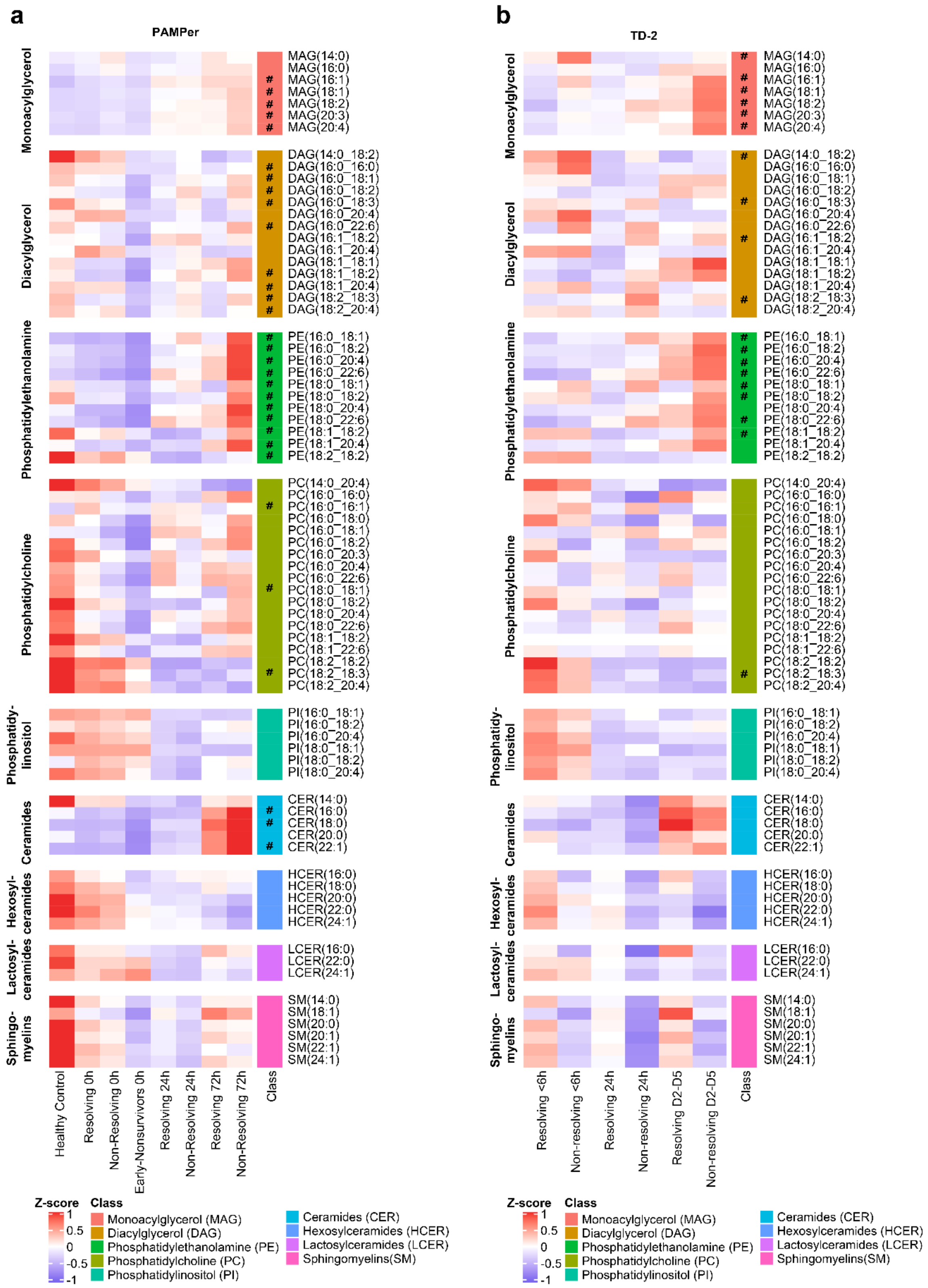

Supplementary Fig.5 Temporal pattern of common lipids of trauma patients from PAMPer and TD-2.

(a-b) Heatmap shows relative levels of 99 common lipid species from 9 major classes across patients. Patients are group by outcome and sampling timepoint. Data comes from PAMPer lipidomics dataset **(A)** or TD-2 untargeted metabolomics dataset **(b)**.

Number sign (#) indicate lipids with log2 fold change >0.4 between non-resolving and resolving trauma patients at 72h (a); non-resolving and resolving trauma patients at D2-D5 (b).

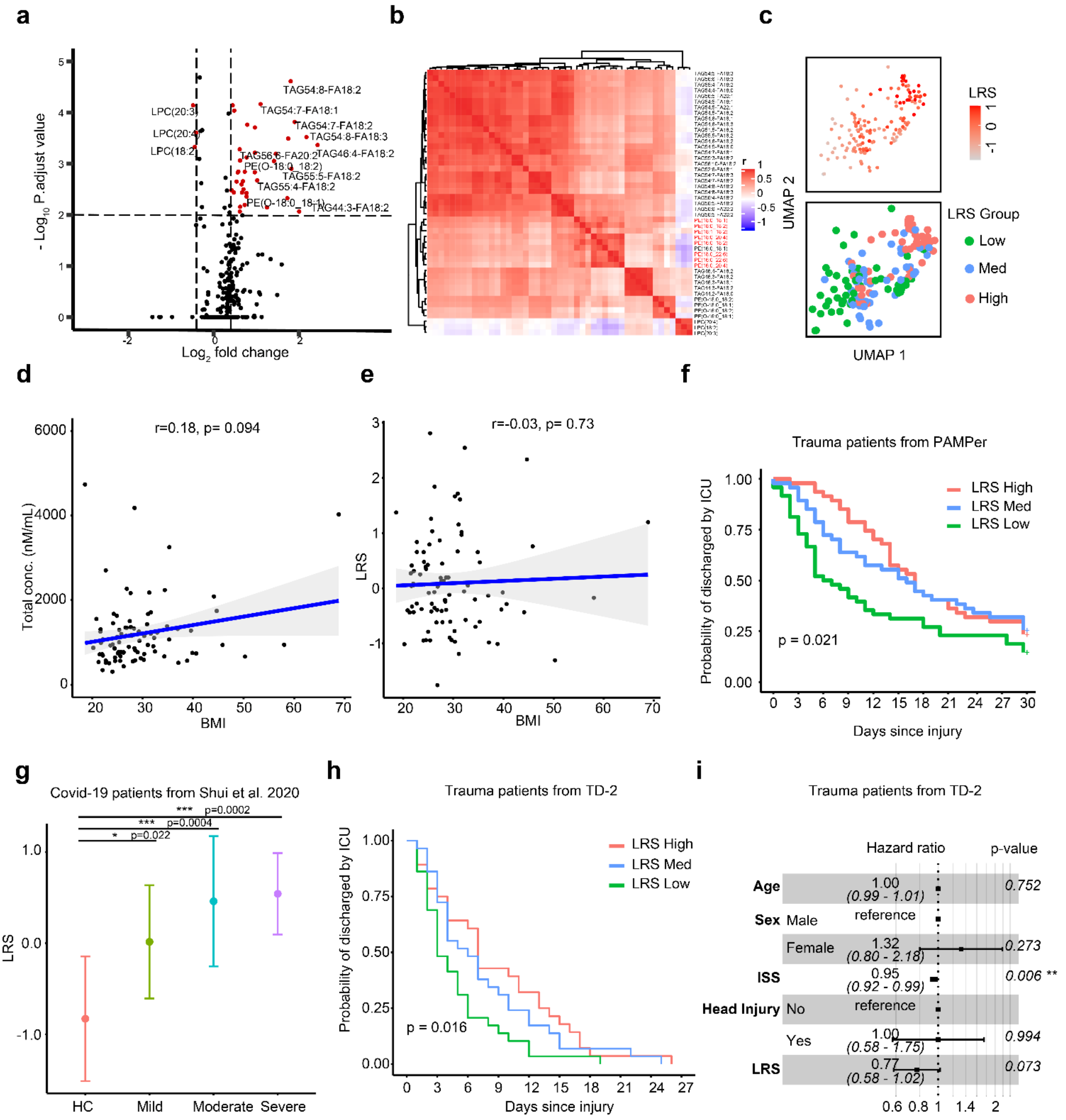

Supplementary Fig.6 Evaluation and external validation of lipid reprogramming score (LRS).

(a) Volcano plot shows the differential lipids in non-resolving patients (n=101) compared to resolving patients (n=41) at 72h after admission. P-values are calculated by using the two-sided Kruskal-wallis test with multiple correction by Benjamini-Hochberg method.

(b) Correlation heatmap of 8 common lipids and 37 selected differential lipids.

(c) UMAP plot of LRS and LRS group among trauma patients surviving at 72h (n=152).

(d-e) Correlation analysis between BMI and total lipid concentration at 72h (D) or LRS (E) in patients (n=89) from PAMPer dataset. Error bands: 95% confidence interval for regression line.

(f) Recovery probability (defined as discharged from intensive care unit) of different LRS groups across days after injury revealed by K-M curve. LRS groups are based on tertiles at 72h after admission for each patient. P-value is calculated by log-rank p test.

(g) Comparison of LRS from patients with COVID-19 from shui et al. Healthy subjects (n=26) and Patients (n=50) are grouped with outcome. Center dots and error bars represent median value, median absolute deviation respectively. Asterisks in (G) indicate statistical significance based on Kruskal-wallis test among 4 group with post-hoc analysis of Dunn test. p value was adjusted by Benjamini-Hochberg method: **, < 0.01.

(h) Recovery probability (defined as discharged by intensive care unit) of different LRS groups across days since injury revealed by K-M curve in TD-2 dataset (n=86). P-value is calculated by log-rank p test.

(i) Forest plot shows the Hazard ratios of clinical factors and LRS score for recovery using cox regression in the TD-2 dataset (n=86). ISS, injury severity score. Error bars: 95% confidence interval.

Source data are provided as a Source Data file.

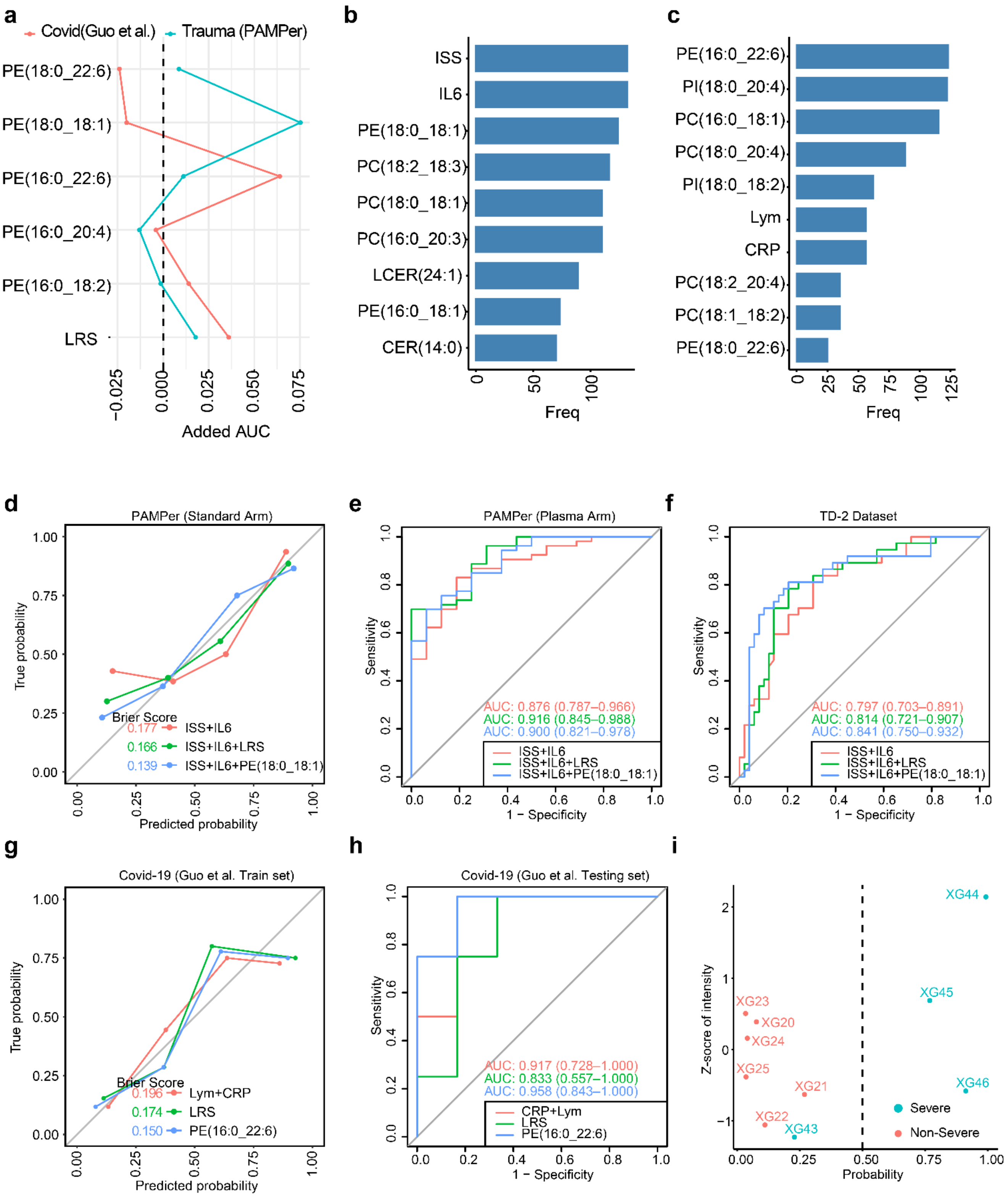

Supplementary Fig.7 Validation of prognostic value for LRS in both trauma and COVID-19

(a) Added AUC from LRS or its components compared to reference model in training set. (Trauma: 73 patients in Standard-of-care arm of PAMPer dataset, Covid-19: 45 patients in train cohort(C1) from Guo. et al.)

(b-c) Frequency of features selected by two-step machine learning approach in patients with trauma (**B**, standard arm of PAMPer, n=73) or Covid-19 (**C**, C1 from guo et al, n=45) (see also methods). Top 10 selected features of all models were shown.

(d) Calibration curve of three prognostic models in 73 patients from standard arm of PAMPer dataset.

(e-f) ROC curve for testing three prognostic models in 69 patients from TP arm of PAMPer dataset (C) or 86 patients from TD-2 dataset(D).

(g) Calibration curve of three prognostic models in 45 patients from the Covid-19 dataset (C1 of Guo et al.)

(h) ROC curve for testing three prognostic signatures in test cohort (C1) from Guo et al of 10 COVID-19 patients.

(i) Performance of the PE (16:0_22:6) in the test cohort (C2) from Guo et al of 10 COVID-19 patients.

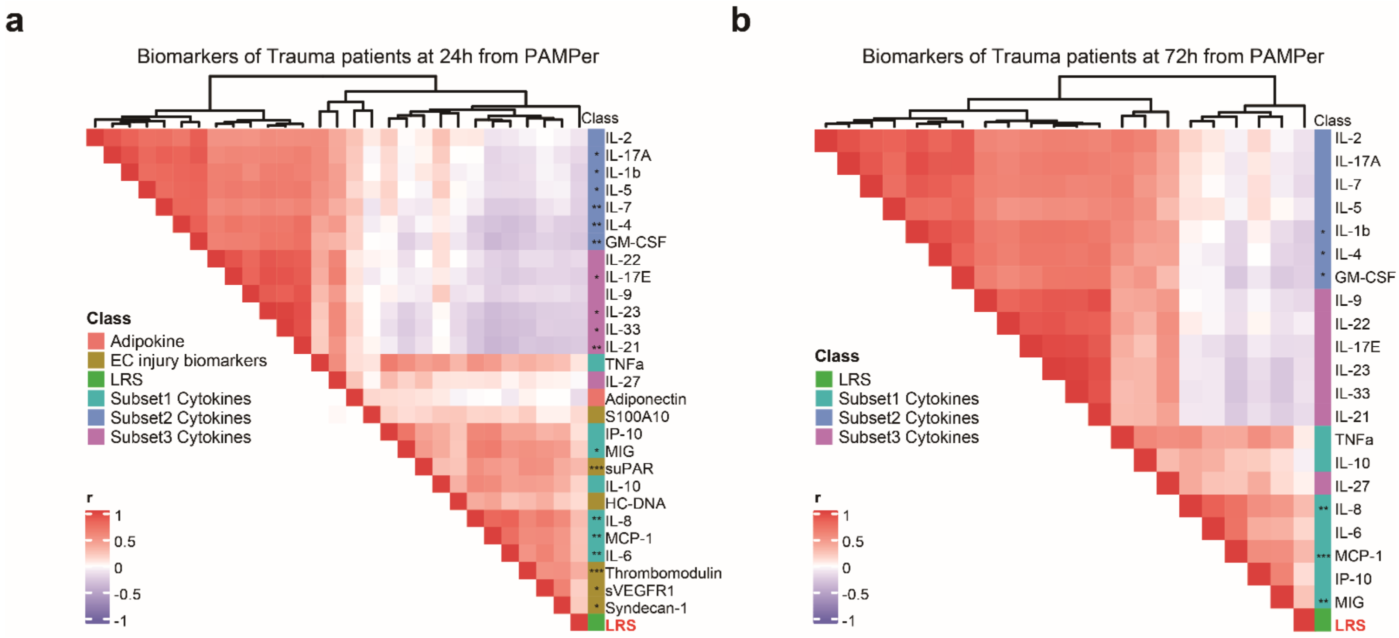

Supplementary Fig.8 Association between LRS and circulating biomarkers or pathways

(a-b) Heatmap shows the correlation between LRS and circulating biomarkers at 24h (n=121) and 72h (n=121) after admission in trauma patients, measured by spearman correlation coefficients.

Asterisks in (A&B) indicate statistical significance for correlation coefficient. Unadjusted p-values are approximated by using the two-sided t distributions: *, < 0.05; **, < 0.01; ***, <0.001.

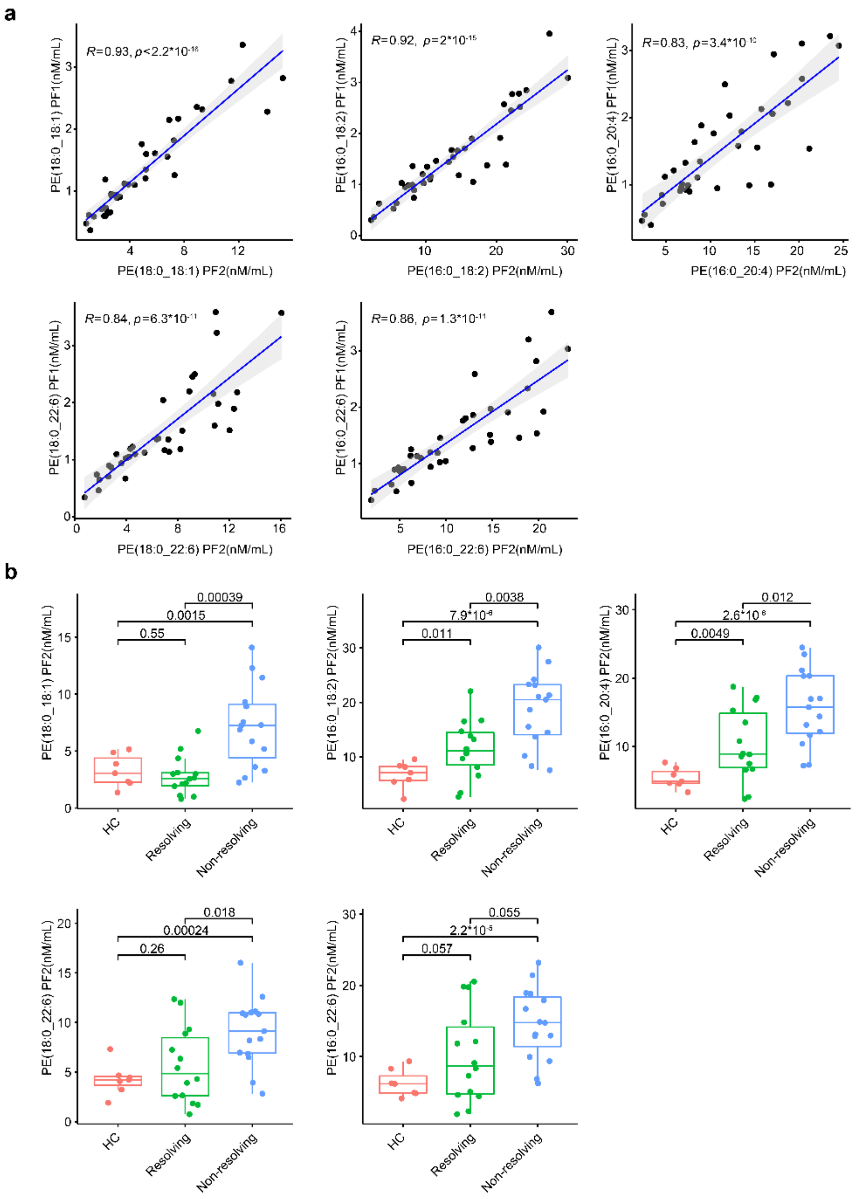

Supplementary Fig.9 Technical Validation of 5 PE species for construction of LRS.

a) Correlation of 5 PE species from LRS between two technical platforms (PF1, PF2: see also methods) from selected subjects (8 Non-fasting healthy controls, 29 Trauma patients at 72h timepoint). R: Pearson correlation coefficient. Error bands: 95% confidence interval for regression line. P-values are approximated by using the two-sided t distributions. **b)** Comparison of the plasma concentrations of 5 PE species of PF2 in healthy controls (HC) (n=7, one outlier was excluded), resolving trauma patients at 72h (n=14), Non-resolving trauma patients at 72h (n=15). P-value were calculated by two-sided student t-test between two groups. Box and whisker plots represent mean value, standard deviation, maximum and minimum values, and individual datapoints.Source data are provided as a Source Data file.

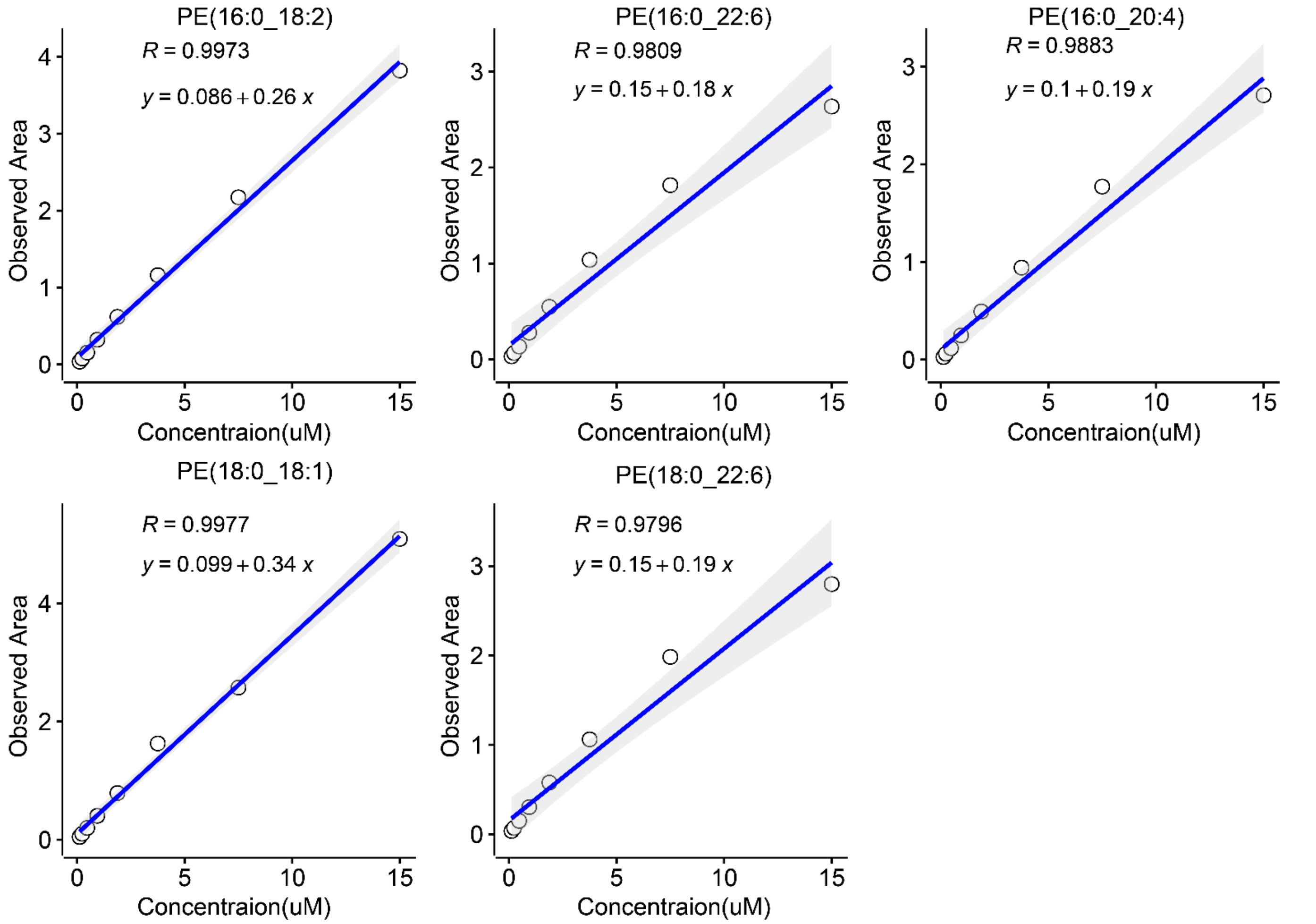

Supplementary Fig.10 Calibration curves of 5 PE species by platform of targeted lipidomics.

Calibration curves of PE(16:0_18:2),PE(16:0_22:6),PE(16:0_20:4),PE(18:0_18:1),PE(18:0_22:6) by platform 2(High-resolution LC-HRMS). Concentration of internal standard was plot by x-axis and its peak size area by LC-HRMS was plot by y-axis. Equation of linear regression and its coefficient was labeled in each panels.

Supplementary Acknowledgement:

The PAMPer study group Investigators and Collaborators

University of Pittsburgh, Presbyterian Hospital; Clinical Coordinating center and enrolling site: Jason L. Sperry, MD, MPH, Francis X. Guyette, MD, MPH, Mazen S. Zenati, MD, PhD, Joshua B.Brown, MD, MSc, Mark H. Yazer, MD, Darrell J. Triulzi, MD Barbara J. EarlyYoung, BSN, Peter W.Adams, BS, Louis H. Alarcon, MD, Clifton W. Callaway, MD, PhD, Brian S. Zuckerbraun, MD, Matthew D. Neal, MD, Raquel M. Forsythe, MD, Timothy R. Billiar, MD, Donald M. Yealy, MD, Andrew B. Peitzman, MD, Meghan L. Buck, Ashley M. Ryman, Elizabeth A. Gimbel, Erin G. Gilchrist, Meghan Buhay, Chung-Chou H. Chang, Victor B. Talisa, Tianyuan Xu, Multidisciplinary Acute Care Research Organization (MACRO)-MACRO Research Specialists, MACRO Clinical Trials Research Associates.

University of Tennessee Health Science Center: Brian J. Daily, MD, Kyle Kalloway, Andrew Yates, Susan Rawn.

Vanderbilt University Medical Center: Richard S. Miller, MD, Judith M.Jenkins.

University of Louisville: Brain G. Harbrecht, MD, Laura S. Trachtenberg, Randi K. Eden.

MetroHealth Medical Center/Case Western Reserve University: Jeffrey A. Claridge, MD, Joanne Fraifogl, Craig Bates, MD.

University of Texas Southwestern/Parkland Memorial Hospital: Herb A.Phelan, MD, Christina Howard, Cari Stebbins.

Texas Health Harris Methodist Hospital: William R.Witham, MD, Cathy McNeill.

University of Pittsburgh Medical Center, Altoona Hospital: A. Tyler. Putnam, MD, Amy Snyder, Jason Ropp.

John Peter Smith Health Network: Therese M. Duane, MD,Celeste Caliman, Mieshia Beamon.
