# Supplementary material for: Lipidomic signatures align with inflammatory patterns and outcomes in critical illness
Source: Nat Commun. 2022 Nov 10;13:6789. doi: 10.1038/s41467-022-34420-4 (PMC9647252; doi:10.1038/s41467-022-34420-4)
Supplement: Supplementary file 3 — Description of Additional Supplementary Files [file 41467_2022_34420_MOESM3_ESM.docx]

**Description of Additional Supplementary Files**

File Name: Supplementary Data 1

Description: Comorbidity and meditation for patients involved in lipidomics measurement of PAMPer trial

File Name: Supplementary Data 2

Description: Statistics results for 29 common species among 4 datasets from trauma or Covid-19 patients

File Name: Supplementary Data 3

Description: Ranking of lipid species between non-resolving and resolving trauma patients at 72h

File Name: Supplementary Data 4

Description: Characteristics of the patients surviving at 72h after admission by LRS group

File Name: Supplementary Data 5

Description: Relationship of differentially lipids species across four trauma and Covid-19 datasets

File Name: Supplementary Data 6

Description: Sensitivity analysis of LRS generation by different species of PE

File Name: Supplementary Data 7

Description: Performance of prognostic value for LRS and individual PE species in Trauma and COVID-19

File Name: Supplementary Data 8

Description: Summary of median relative standard deviation (RSD) values in 14 classes of metabolomics assay

File Name: Supplementary Data 9

Description: Characteristics of the matched male patients for technical validation of 5 PE species

File Name: Supplementary Data 10

Description: List of internal standards and their concentration in global lipidomic platform

File Name: Supplementary Data 11

Description: Raw lipidomic dataset of 17 healthy subjects and 193 trauma patients of PAMPer trial
