# Supplementary material for: Lipidomic signatures align with inflammatory patterns and outcomes in critical illness
Source: Nat Commun. 2022 Nov 10;13:6789. doi: 10.1038/s41467-022-34420-4 (PMC9647252; doi:10.1038/s41467-022-34420-4)
Supplement: Supplementary file 15 — Reporting Summary [file 41467_2022_34420_MOESM15_ESM.pdf]

nature research

Corresponding author(s): Timothy R. Billiar, MD

Last updated by author(s): Aug 28, 2022

Reporting Summary

Nature Research wishes to improve the reproducibility of the work that we publish. This form provides structure for consistency and transparency in reporting. For further information on Nature Research policies, see our Editorial Policies and the Editorial Policy Checklist.

Statistics

For all statistical analyses, confirm that the following items are present in the figure legend, table legend, main text, or Methods section.

n/a	Confirmed	
☐	☒	The exact sample size (*n*) for each experimental group/condition, given as a discrete number and unit of measurement
☐	☒	A statement on whether measurements were taken from distinct samples or whether the same sample was measured repeatedly
☐	☒	The statistical test(s) used AND whether they are one- or two-sided<br>*Only common tests should be described solely by name; describe more complex techniques in the Methods section.*
☐	☒	A description of all covariates tested
☐	☒	A description of any assumptions or corrections, such as tests of normality and adjustment for multiple comparisons
☐	☒	A full description of the statistical parameters including central tendency (e.g. means) or other basic estimates (e.g. regression coefficient) AND variation (e.g. standard deviation) or associated estimates of uncertainty (e.g. confidence intervals)
☐	☒	For null hypothesis testing, the test statistic (e.g. *F*, *t*, *r*) with confidence intervals, effect sizes, degrees of freedom and *P* value noted<br>*Give P values as exact values whenever suitable.*
☒	☐	For Bayesian analysis, information on the choice of priors and Markov chain Monte Carlo settings
☐	☒	For hierarchical and complex designs, identification of the appropriate level for tests and full reporting of outcomes
☐	☒	Estimates of effect sizes (e.g. Cohen's *d*, Pearson's *r*), indicating how they were calculated

Our web collection on statistics for biologists contains articles on many of the points above.

Software and code

Policy information about availability of computer code

Data collection	Lipidomics dataset is generated by LC-MS/MS from Metabolon Inc.
Data analysis	Code supporting the current study is deposited at https://github.com/Junru-max/PAMPer-Lipidomic-analysis.<br>Key open-sourced packages in R (Version 3.6.0) used in this manuscript :<br>ComplexHeatmap_2.5.2 Hmisc_4.4.1

For manuscripts utilizing custom algorithms or software that are central to the research but not yet described in published literature, software must be made available to editors and reviewers. We strongly encourage code deposition in a community repository (e.g. GitHub). See the Nature Research guidelines for submitting code & software for further information.

Data

Policy information about availability of data

All manuscripts must include a data availability statement. This statement should provide the following information, where applicable:

- Accession codes, unique identifiers, or web links for publicly available datasets
- A list of figures that have associated raw data
- A description of any restrictions on data availability

The lipidomics dataset generated in this study have been deposited in the Mendeley Data under DOI 10.17632/7stf7dtxcz.2 (https://data.mendeley.com/datasets/7stf7dtxcz/draft?a=3e078e7f-5068-4b8e-a5a9-ef414db279bd) and are provided in Supplementary Data11. The individual internal standard of Plasma lipidomic profiling by Metabolon Inc (Morrisville, NC 27560, USA) is commercially available(https://sciex.com/products/consumables/lipidyzer-platform-kits) and can

be found in Supplementary Data10. The public metabolomic or lipidomic dataset re-used in this study can be found at https://doi.org/10.1016/j.cell.2020.05.032 and https://doi.org/10.1016/j.cmet.2020.06.016. The remaining data are available within the article or from the authors upon request.

Field-specific reporting

Please select the one below that is the best fit for your research. If you are not sure, read the appropriate sections before making your selection.

- [x] Life sciences
- [ ] Behavioural & social sciences
- [ ] Ecological, evolutionary & environmental sciences

For a reference copy of the document with all sections, see nature.com/documents/nr-reporting-summary-flat.pdf

Life sciences study design

All studies must disclose on these points even when the disclosure is negative.

Sample size

For the raw PAMPer trail, We estimated that enrollment of 530 prehospital patients would result in 504 eligible patients with complete data and would provide the trial with 88% power to detect a difference of 14 percentage points (8.0% vs. 22.0%) in 30-day mortality between the plasma group and the standardcare group, on the basis of published mortality estimates.
For the omics study, we try to match the overall characteristic of raw PAMPer trail due to the unavailability for the subset of blood samples. The detailed workflow can be seen in supplementary figure 1. For group-wise comparison among patients, we examined on those had at least 40 individuals or events per group (Injury severity, treatments arms, Outcome et al.) and used statistical analyses to take the sample size into account. The sample size is confirmed sufficient by using a stringent significant threshold in downstream statistics.

Data exclusions

No samples from patients or subjects were identified as outlier to be excluded.

Replication

The quality control sample was generated by combining a small aliquot from the entire set of samples into a single pooled CMTX (ClientSample matrix). Four aliquots of the CMTX were run on each plate of 36 samples. One each was injected at the beginning and end of the run, with the other two roughly evenly spaced between the remaining samples. The internal standard was run multiple times throughout the experiment. Instrument variability was evaluated by calculating median relative SD (RSD) from the quality control sample matrix. The median RSD value is less than 4%. The median RSD values for 14 lipid classes can be found in Table S8.

Randomization

Randomization for raw PAMPer trail was described in previous study(Sperry et al. NEJM. Published 26th July, 2018. N Engl J Med 2018;379:315-26.DOI: 10.1056/NEJMoa1802345).
For this study, randomization was arranged by computing in R for omics measurement.

Blinding

Each subject and sample was labeled with a randomized ID whose annotation and clinical status were kept blinded during data collection and analyses.

Reporting for specific materials, systems and methods

We require information from authors about some types of materials, experimental systems and methods used in many studies. Here, indicate whether each material, system or method listed is relevant to your study. If you are not sure if a list item applies to your research, read the appropriate section before selecting a response.

Materials & experimental systems

n/a	Involved in the study	
x		Antibodies
x		Eukaryotic cell lines
x		Palaeontology and archaeology
x		Animals and other organisms
	x	Human research participants
x		Clinical data
x		Dual use research of concern

Methods

n/a	Involved in the study	
x		ChIP-seq
x		Flow cytometry
x		MRI-based neuroimaging

Human research participants

Policy information about studies involving human research participants

Population characteristics	193 trauma patients from PAMPer trial with 17 healthy subjects were involved in this study. More information were provided in Methods and Table1.
Recruitment	Detailed information of recruitment of raw PAMPer trial can be seen in our previous study (Sperry et al. NEJM. Published 26th July, 2018. N Engl J Med 2018;379:315-26.DOI: 10.1056/NEJMoa1802345). Patients selection of sampling and lipidomic measurement can be seen in supplementary Figure 1. Of the 523 patients involved in the analysis of PAMPer trial, only 292 patients with blood sample were avaible for the lipidomic analysis. All samples of non-survivors (n=83) were kept. 1:1 match was used in survived patients with/without traumatic brain injury in both arms and 99 patients were excluded. Finally, 193 patients with blood samples were selected for the global lipidomic analysis.
Ethics oversight	PAMPer trial was approved by the IRB of University of Pittsburgh as previously described19. The Emergency Exception from Informed Consent (EFIC) protocol from the Human Research Protection Office of the US Army Medical Research and Material Command was applied to this study. Further details of emergency exception to informed consent can be found at our official website of PAMPer trial at https://crisma.upmc.com/apps/PAMPer/home/.Registered information and detailed study protocol are available on https://clinicaltrials.gov/ct2/show/NCT01818427. All participants or their legally authorized representatives provided the consent to continue participation. No participant compensation was involved in the study. Healthy volunteers were enrolled in an observational study approved by the University of Pittsburgh Institutional Review Board (PRO08010232). The detailed study protocol is available on https://www.clinicaltrials.gov/ct2/show/NCT00250523. Written informed consent was obtained from all the subjects. No participant compensation was involved in the study.

Note that full information on the approval of the study protocol must also be provided in the manuscript.
